# A case of branchial cleft cyst in the esophagus: A case report

**DOI:** 10.1097/MD.0000000000045429

**Published:** 2025-11-07

**Authors:** Yiping Han, Xiaoli Zuo, Yaqi Mi

**Affiliations:** a Qingdao Fifth People’s Hospital Affiliated to Qingdao University, Qingdao, Shandong Province, China; b Qingdao Hospital of Traditional Chinese Medicine, Qingdao, Shandong Province, China.

**Keywords:** branchial cyst, esophagus, pathological character, treatment and outcome

## Abstract

Branchial cleft cyst, also known as a benign lymphoepithelial cyst, is a congenital disorder with an unclear pathogenesis. It most commonly occurs in the anterior triangle of the neck along the upper third of the sternocleidomastoid muscle, while occurrences at other sites are exceedingly rare. Diagnosis through ultrasound, computed tomography, or magnetic resonance imaging is challenging. Endoscopic submucosal dissection (ESD) enables the acquisition of complete specimens, aiding in the diagnosis and treatment of such lesions. This study presents a case of an esophageal branchial cleft cyst that was successfully diagnosed and treated with ESD, resulting in an excellent postoperative outcome. This case enhances clinical recognition of this rare entity and demonstrates the therapeutic utility of ESD in its management.

## 1. Introduction

Branchial cleft cysts are developmental cysts of unknown etiology. The prevailing theory suggests their origin from remnants of the 1st to 4th branchial arches.^[1,2]^ Subsequent studies, noting the presence of lymphoid tissue in most cyst specimens, proposed the “benign lymphoepithelial cyst theory,” classifying them as cystic lymphadenopathy or benign lymphoepithelial cysts.^[3]^ Thus, the terms “branchial cleft cyst” and “lymphoepithelial cyst” are often used interchangeably.^[4]^ Branchial cleft cysts are categorized into 4 types based on their origin. The 2nd branchial cleft cyst is the most common, accounting for approximately 95% of cases, typically located in the upper 3rd of the anterior cervical triangle. The 1st and 3rd types are less frequent, while the 4th type, originating near the recurrent laryngeal nerve, is exceptionally rare.^[1,5]^ This paper reports a case of a 4th branchial cleft cyst in the esophagus, diagnosed and successfully treated via endoscopic submucosal dissection (ESD). The clinicopathological features, diagnostic approach, and treatment are discussed to further elucidate this rare condition.

## 2. Case report

**Figure 1.**
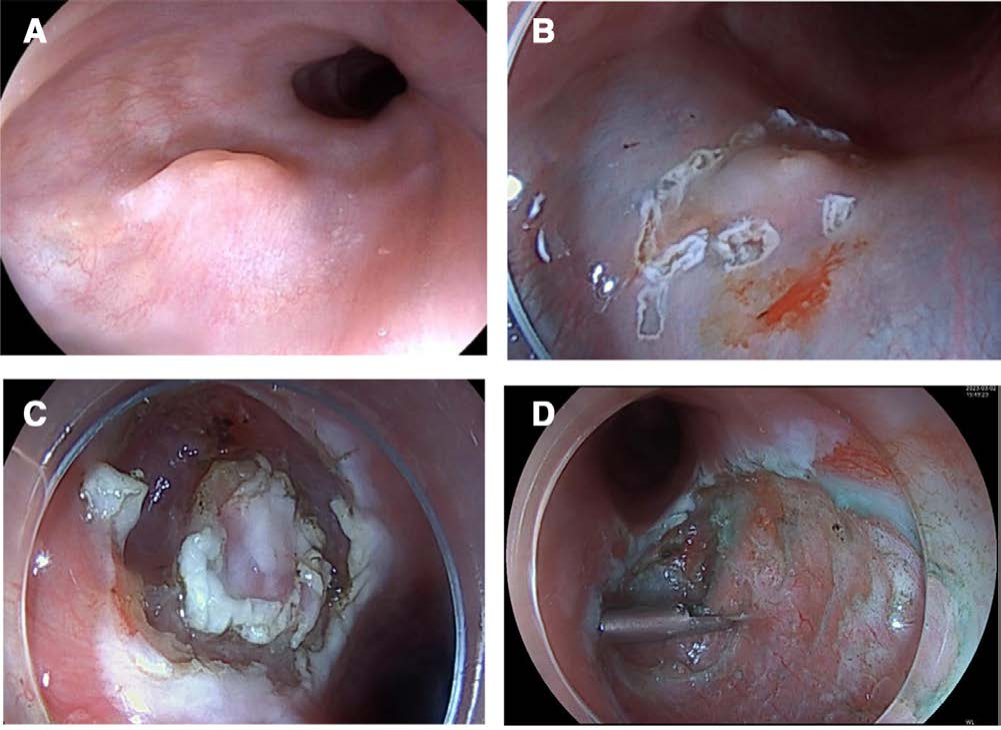
Endoscopic imaging. (A) The tumor was located in the submucosa of the esophagus about 20 cm from the incisor, about 6 × 6 mm; (B and C) sodium hyaluronate was injected into the submucous membrane around the tumor, and the mucosa of the lateral margin of the tumor was incised with a mucotomy knife. (D) The tumor was completely removed into the submucosa, and the musculature clipped by hemostatic forceps was locally damaged.

A 47-year-old male presented with persistent symptoms of acid regurgitation, heartburn, and hiccups, alongside occasional dysphagia-associated retrosternal discomfort, which had been recurring for over a year. The symptoms persisted despite empirical therapy with omeprazole and hydrotalcite chewable tablets. His past medical history was significant for hypertension and lumbar disc herniation. Family history was noncontributory. Physical examination was notable only for mild epigastric tenderness upon palpation; no other abnormalities were detected. Following admission, a series of investigations were performed. The lipid profile revealed hypertriglyceridemia (2.42 mmol/L) and a borderline elevated total cholesterol (5.48 mmol/L), with an LDL level of 3.74 mmol/L. The patient was also found to have hyperuricemia (476.2 μmol/L) and was positive for hepatitis B surface antigen at 76.44 IU/mL. Complete blood count, liver and renal function tests, electrolytes, and coagulation profiles were all within normal limits. Initial chest computed tomography (CT) revealed chronic bronchitis, bilateral emphysema, pulmonary bullae, and vascular calcifications. Abdominal CT indicated cholecystitis and appendicitis. The initial clinical impression, based on the presenting symptoms and laboratory results, was chronic gastritis. However, due to the lack of clinical response to empiric therapy with a proton-pump inhibitor, an esophagogastroduodenoscopy was arranged to confirm the diagnosis and rule out other pathology. Upper endoscopy showed chronic non-atrophic gastritis and a 6 × 6 mm submucosal protrusion in the esophagus, 20 cm from the incisors. Given that chronic non-atrophic gastritis was deemed insufficient to account for the patient’s clinical manifestations, the esophageal lesion was considered the more likely etiology. To confirm the nature of the lesion, ESD was performed after obtaining informed consent. During the procedure, a submucosal mass was identified and resected en bloc following sodium hyaluronate injection and mucosal incision (Fig. 1).

**Figure 2.**
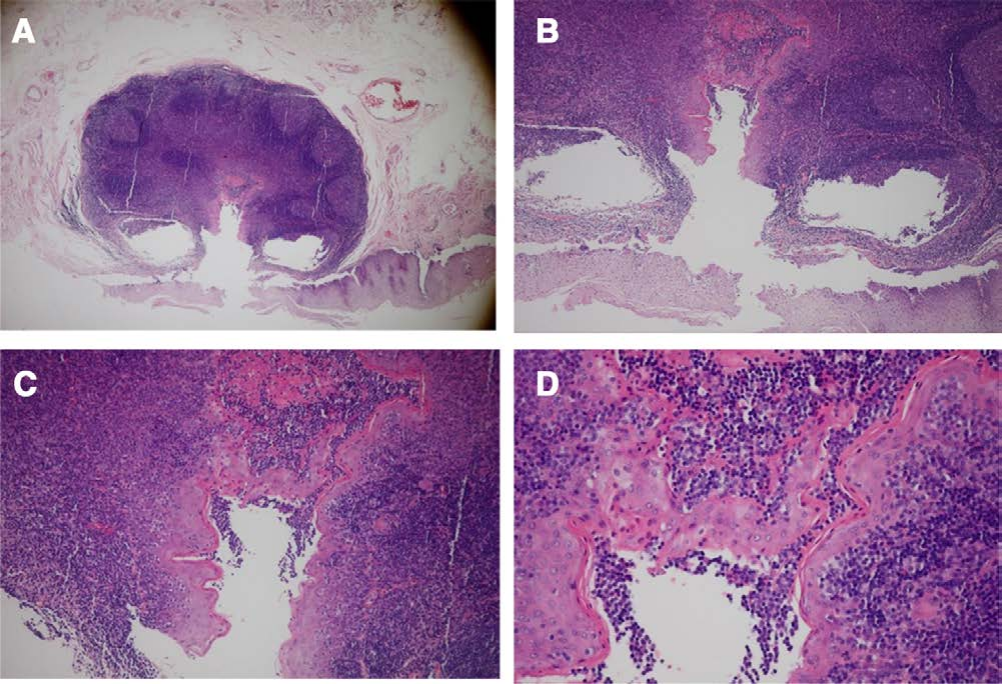
Pathological images. Capsule wall lined multilayer squamous epithelium with atkeratosis. There was a large amount of lymphoid tissue in the cyst wall and lymphoid follicles were formed. (A) Magnification 40×, (B) magnification 100×, (C) magnification 200×, and (D) magnification 400×.

Pathological findings: the cyst wall was lined with stratified squamous epithelium exhibiting keratinization. Abundant lymphoid tissue with follicular formation was observed within the wall (Fig. 2). These findings confirmed the diagnosis of an esophageal branchial cleft cyst.

The definitive diagnosis, confirmed by postoperative histopathological examination of the ESD specimen, was an esophageal branchial cleft cyst. The patient tolerated the procedure well and was discharged without complications. At follow-up, the patient reported complete resolution of all prior symptoms, including acid reflux and retrosternal dysphagia. Surveillance endoscopy is planned for 1 year post-procedure.

## 3. Discussion

Esophageal branchial cleft cysts are exceedingly rare. Their clinical manifestations depend primarily on the cyst’s size and location, as well as the presence or absence of secondary infection. Small cysts that do not cause mass effect or compress surrounding structures may remain asymptomatic for a prolonged period. In the present case, the patient’s clinical presentation was characterized by acid reflux and a persistent retrosternal foreign body sensation. While CT and magnetic resonance imaging are primary diagnostic tools for branchial cleft cysts,^[5]^ their utility is limited for small esophageal lesions, as demonstrated in this case. Endoscopic ultrasonography may aid diagnosis but has shown inconsistencies with pathological results in prior reports.^[1,6]^ Due to their nonspecific endoscopic appearance, esophageal branchial cleft cysts can be difficult to distinguish from other submucosal lesions, such as esophageal leiomyomas or neurogenic tumors, rendering preoperative diagnosis challenging. Consequently, to obtain a definitive histopathological diagnosis (which is considered the gold standard) we proceeded directly with ESD. The lesion was resected en bloc and submitted in its entirety for pathological examination.

Histologically, these cysts are characterized by stratified squamous epithelium with lymphoid infiltrates and follicular formation.^[1,6–8]^ In this pathological examination, the cyst wall was lined with lamellar squamous epithelium accompanied by keratosis. There was a large amount of lymphoid tissue in the cyst wall and the formation of lymphoid follicles was observed, which was consistent with previous reports and further confirmed the pathological diagnostic criteria of this lesion.

Although malignant transformation is rare,^[9]^ surgical intervention is warranted for symptomatic cases, such as those causing dysphagia or hoarseness.^[6]^ In this case, in order to obtain a complete lesion specimen, ESD treatment was performed with the consent of the patient. The pathological diagnosis and treatment of the lesion were confirmed to be esophageal branchial cyst, and the patient recovered well after surgery without recurrence. Ultimately, the patient’s condition (which had been troubling him for over a year) was successfully resolved. We present this rare case to enhance clinical awareness and deepen the understanding of esophageal branchial cleft cysts among gastroenterologists.

## Author contributions

**Conceptualization:** Yaqi Mi.

**Data curation:** Yiping Han.

**Formal analysis:** Yaqi Mi.

**Investigation:** Yiping Han.

**Methodology:** Xiaoli Zuo.

**Supervision:** Yaqi Mi.

**Validation:** Xiaoli Zuo.

**Writing – original draft:** Yiping Han.

**Writing – review & editing:** Yiping Han, Yaqi Mi.
